# Genome-Guided Analysis of Seven Weed Species Reveals Conserved Sequence and Structural Features of Key Gene Targets for Herbicide Development

**DOI:** 10.3389/fpls.2022.909073

**Published:** 2022-06-29

**Authors:** Sarah Shah, Thierry Lonhienne, Cody-Ellen Murray, Yibi Chen, Katherine E. Dougan, Yu Shang Low, Craig M. Williams, Gerhard Schenk, Gimme H. Walter, Luke W. Guddat, Cheong Xin Chan

**Affiliations:** ^1^Australian Centre for Ecogenomics, School of Chemistry and Molecular Biosciences, The University of Queensland, Brisbane, QLD, Australia; ^2^School of Chemistry and Molecular Biosciences, The University of Queensland, Brisbane, QLD, Australia; ^3^School of Biological Sciences, The University of Queensland, Brisbane, QLD, Australia; ^4^Sustainable Minerals Institute, The University of Queensland, Brisbane, QLD, Australia; ^5^Australian Institute for Bioengineering and Nanotechnology, The University of Queensland, Brisbane, QLD, Australia

**Keywords:** genetics, herbicide resistance, weed genomics, sequence analysis, gene targets, protein targets, acetolactate synthase, EPSP synthase

## Abstract

Herbicides are commonly deployed as the front-line treatment to control infestations of weeds in native ecosystems and among crop plants in agriculture. However, the prevalence of herbicide resistance in many species is a major global challenge. The specificity and effectiveness of herbicides acting on diverse weed species are tightly linked to targeted proteins. The conservation and variance at these sites among different weed species remain largely unexplored. Using novel genome data in a genome-guided approach, 12 common herbicide-target genes and their coded proteins were identified from seven species of Weeds of National Significance in Australia: *Alternanthera philoxeroides* (alligator weed), *Lycium ferocissimum* (African boxthorn), *Senecio madagascariensis* (fireweed), *Lantana camara* (lantana), *Parthenium hysterophorus* (parthenium), *Cryptostegia grandiflora* (rubber vine), and *Eichhornia crassipes* (water hyacinth). Gene and protein sequences targeted by the acetolactate synthase (ALS) inhibitors and glyphosate were recovered. Compared to structurally resolved homologous proteins as reference, high sequence conservation was observed at the herbicide-target sites in the ALS (target for ALS inhibitors), and in 5-enolpyruvylshikimate-3-phosphate (EPSP) synthase (target for glyphosate). Although the sequences are largely conserved in the seven phylogenetically diverse species, mutations observed in the ALS proteins of fireweed and parthenium suggest resistance of these weeds to ALS-inhibiting and other herbicides. These protein sites remain as attractive targets for the development of novel inhibitors and herbicides. This notion is reinforced by the results from the phylogenetic analysis of the 12 proteins, which reveal a largely consistent vertical inheritance in their evolutionary histories. These results demonstrate the utility of high-throughput genome sequencing to rapidly identify and characterize gene targets by computational methods, bypassing the experimental characterization of individual genes. Data generated from this study provide a useful reference for future investigations in herbicide discovery and development.

## Introduction

The management and control of weed species remain a major global problem, largely due to increased frequencies of herbicide resistance and lack of new herbicides entering the market. Weeds threaten the environment by damaging the natural landscapes and waterways, leading to displacement of native species, degradation of the ecosystem, and reduction in biodiversity. In Australia, over 30 Weeds of National Significance (Atlas of Living Australia, 2022) have been identified to date, due to the negative impacts they have on the environment, economy, and society. These weeds were selected based on their invasiveness and ability to spread and persist in natural and agricultural environments, and the severity of the risks they pose. Legislation has been implemented to contain their spread by mandating reporting and removal. In 2018, the financial cost of weed control across natural agriculture and non-agriculture sectors (e.g., management of environments and indigenous lands) was estimated between $4.5 and $6.0 billion per annum in Australia (McLeod, 2018).

Application of herbicides is one of the most common approaches for managing weeds. Other approaches include manual/mechanical removal and biological control using insects and plant pathogens. Increasingly, combinations of these approaches are used in an integrated pest management framework. However, the use of herbicides, which is often more cost-effective and less labor-intensive, remains highly popular. Two of the most widely used commercial herbicides are the acetolactate synthase [ALS, also known as acetohydroxyacid synthase (AHAS)] inhibitors, and glyphosate (Funke et al., 2006; Garcia et al., 2017). ALS is the first enzyme in the branched-chain amino acid (BCAA) biosynthesis pathway that yields valine, isoleucine and leucine (Garcia et al., 2017), whereas glyphosate inhibits 5-enolpyruvylshikimate-3-phosphate (EPSP) synthase that is central to the biosynthesis pathway of aromatic amino acids (Funke et al., 2006). It is most commonly assumed that ALS inhibitors function by preventing substrate from gaining access to the active site of the enzyme. However, it has also been proposed that inhibition of ALS could also result in the accumulation of 2-keto-butyrate, a substrate for ALS known to be toxic to plants (Dayan and Duke, 2020).

A sound understanding of the specificity and effectiveness of herbicides at targeting weed species is critical for selecting or designing target-specific herbicides with optimized potency. The risk of weeds developing resistance should also be minimized along with potential negative impact on non-target species and the broader environment. An intimate knowledge of metabolic pathways linked to herbicide action will guide targeted prediction of herbicide efficiency for specific weeds. Genome and proteome data from weed species present a powerful resource for identifying inherent variations in gene and protein sequences (e.g., membrane transporters and cytochrome P_450_ enzymes) that may influence and impinge upon the effectiveness of specific herbicides, and for identifying genes or proteins conferring herbicide resistance (Ravet et al., 2018; Fonseca et al., 2020; Garcia et al., 2020). To date, however, little genome-scale data are available from invasive or problematic weed species, presenting a significant knowledge gap. Problematic weed species are highly diverse and classified in various Orders, distinct from the model weed plant of *Arabidopsis thaliana*, thus observations based on analysis on *Arabidopsis* may not be generalizable to other species.

In this study, we adopted a genome-guided approach to assess 12 common herbicide-target genes and their coded proteins among seven invasive weed species in Australia. Novel genome data generated from these species enabled the intersection of sequence and protein structural information to elucidate target sites for glyphosate and ALS inhibitors, and for potential development of other herbicides.

## Materials and Methods

### Weed Species and Collection of Leaf Material

**FIGURE 1:**
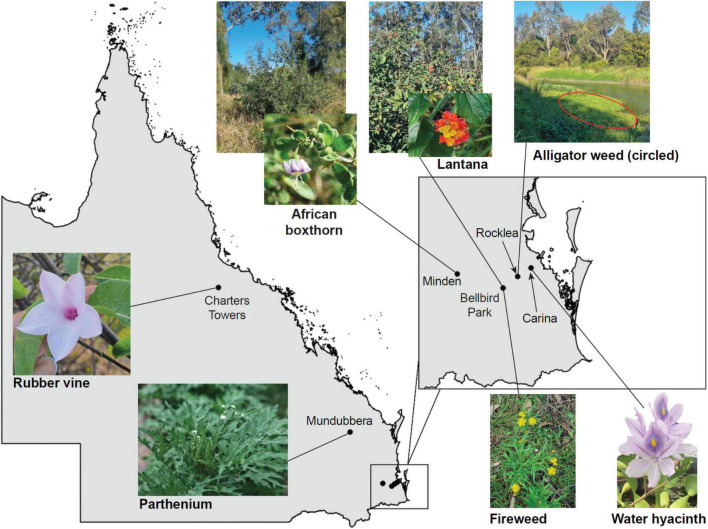
Locations in Queensland, Australia, where plant material or seeds (to grow plants for leaf samples) were collected from the Weeds of National Significance investigated in this study. Clockwise from left: Rubber vine (*Crytostegia grandiflora*) in Charters Towers, pictured in flower; African Boxthorn (*Lycium ferocissimum*) infestation from Minden, with flower inset; lantana (*Lantana camara*) infestation at Bellbird Park, with flower inset; alligator weed (*Alternanthera philoxeroides*) infestation (and other weeds) in Stable Swamp Creek, Rocklea; water hyacinth (*Eichhornia crassipes*, also known as *Pontederia crassipes*) from Bulimba Creek, Carina (photo by Jennifer Evans and used with permission); fireweed (*Senecio madagascariensis*) from Bellbird Park, in flower; and parthenium (*Parthenium hysterophorus*) from Mundubbera.

Seven species of Weeds of National Significance were chosen for this study (Figure 1 and Table 1), based on their particularly pernicious role in the destruction of the Australian environment and their impact on agricultural yields. For example, lantana forms dense thickets that smother native vegetation (Gooden et al., 2009) and is potentially poisonous to livestock (Sharma et al., 2007; Ntalo et al., 2022), whereas alligator weed impacts both aquatic and terrestrial environments and can displace native flora, especially along river banks (Sainty et al., 1997). Leaf material for each weed species was collected from field infestations in Queensland or plants in culture (Table 1), and transported to the University of Queensland (Saint Lucia campus, Brisbane) for subsequent analysis.

**TABLE 1:** Seven weed species used in this study.

Common name	Species name	Order	Leaf collection site
Alligator weed	*Alternanthera philoxeroides*	Caryophyllales	Rocklea, Queensland
African boxthorn	*Lycium ferocissimum*	Solanales	Minden, Queensland
Fireweed	*Senecio madagascariensis*	Asterales	Bellbird Park, Queensland
Lantana	*Lantana camara*	Lamiales	Bellbird Park, Queensland
Parthenium	*Parthenium hysterophorus*	Asterales	Lab cultures (Queensland Department of Agriculture and Fisheries, Ecoscience Precinct, Brisbane); grown from seeds collected at Mundubbera, Queensland
Rubber vine	*Cryptostegia grandiflora*	Gentianales	Lab cultures (Univeristy of Queensland), grown from seeds acquired from Tropical Weeds Research Centre, Charters Towers, Queensland
Water hyacinth	*Eichhornia crassipes* (also known as *Pontederia crassipes*)	Commelinales	Carina, Queensland

Each field sample was identified by reviewing morphology and habit of the plant against the diagnostic features outlined in the Lucid Key Server for Environmental Weeds of Australia.^1^ Leaf samples were cut from plants, placed inside paper seed packets, and kept cool using ice bricks inside polystyrene boxes until delivery. The leaves were cleaned with reverse-osmosis water, snap-frozen in liquid nitrogen and stored at −80°C until DNA extraction.

### Generation of Genome Data, *de novo* Genome Assembly, and Gene Prediction

For each weed species, genomic DNA was extracted from the leaf tissue using the ISOLATE II Plant DNA Kit (Meridian Bioscience, Cincinnati, OH, United States) following the manufacturer’s manual. Integrity of the extracted DNA was confirmed by agarose gel electrophoresis (Supplementary Figure 1), and DNA quality was assessed using Nanodrop 2000 Spectrophotometer (ThermoFisher Scientific, Waltham, MA, United States). All seven DNA samples (2–4 μg each; 20–60 mg/mL) were sent to the Australian Genome Research Facility (Melbourne, Australia) for preparation of sequencing libraries (Illumina TruSeq; average insert size 445 bp) and sequencing using the NovaSeq 6000 platform (2 × 150 bp paired-end, multiplexed in a single S4 flow cell); base calling was conducted using llumina bcl2fastq version 2.20.0.422. In total, 928.2 Gbp of sequence data were generated from the seven species (Supplementary Table 1). All sequence data were processed using fastp v0.20.0 (Chen et al., 2018) at default setting to remove adapter sequences, poly-G fragments (a known artifact in NovaSeq 6000 data), and remaining sequence reads of length < 50bp; this yielded 895.5 Gbp of filtered data (Supplementary Table 1). For each dataset, a haploid genome size was estimated based on the distribution of *k*-mer coverage (*k* = 21) generated using Jellyfish v2.3.0 (Marçais and Kingsford, 2011) adapting the *in silico* approach from earlier studies (Lin et al., 2015; González-Pech et al., 2021). Briefly, a haploid genome size is estimated by dividing the number of all observed *k*-mers by the fold-coverage of *k*-mers at the first peak of the frequency distribution. All sequence data generated from this study are available on NCBI Sequence Read Archive (BioProject accession PRJEB47218).

For each species, using the corresponding filtered reads, MaSuRCA v3.3.3 (Zimin et al., 2013) was used to assemble the genome *de novo* into contiguous sequences (i.e., genome scaffolds). Sequences that exhibit unusual G + C content and genome-scaffold lengths were deemed putative contaminant sequences and were removed following Chen et al. (2020), yielding the final genome assembly (i.e., the representative, primary genome assembly). Benchmark Universal Single-Copy Orthologs (BUSCO) v4.1.2 (Waterhouse et al., 2018) were used to assess the completeness of each assembled genome, based on recovery of core conserved genes in the viridiplantae_odb10 dataset (at *genome* mode). The assembled genome of alligator weed is highly fragmented (1.16 million scaffolds, N50 length 3.3 Kbp) with low BUSCO recovery (47.0%; Supplementary Table 2). Different tools are expected to yield different genome assemblies in which some sequence regions are better resolved in one compared to another. To maximize recovery of genome data from alligator weed, the data were also assembled *de novo* using another common tool, CLC Genomics Workbench v10 (Qiagen); this CLC assembly is considered as secondary genomic evidence for alligator weed. For each primary genome assembly from the seven weed species, MAKER v2.31.10 (Cantarel et al., 2008; Holt and Yandell, 2011) was used to predict protein-coding genes, guided by the manually curated protein sequences in SwissProt release 02032020. All assembled genomes, and the predicted gene and protein sequences are available at https://doi.org/10.48610/c22c10c.

### Identification of 18S Ribosomal RNA Sequences for Species Validation

From each genome dataset, full-length 18S rRNA sequences were identified using hmmscan v3.2.1 (Eddy, 2011) with a hidden Markov model profile created using reference 18S rRNA sequences from 1,897 angiosperm species (Maia et al., 2014), and a BLASTn v2.10.1+ (Altschul et al., 1990) search using this reference as query. The 18S rRNA sequences were recovered from the pimary genome assemblies of all weed species except for alligator weed, in which the more-complete 18S rRNA sequence was recovered from the CLC assembly.

### Identification and Analysis of Herbicide-Targeting Genes

We focused on 12 key genes that are known or are potential targets for commercial herbicides (i.e., glyphosate and ALS inhibitors) (Table 2). Of these, the catalytic subunit (CSU) of ALS is the target for ALS inhibitors. The gene encoding EPSP synthase is the target for glyphosate, whereas genes encoding acetyl-CoA carboxylase (ACCase) and D1 protein of photosystem II are targets for other herbicides. In all cases the presence of these genes is essential for survival of plants.

**TABLE 2:** The 12 genes analyzed in this study.

Gene name	Gene product (protein)	Herbicide targeting the gene or the implicated molecular function
*ALS* (CSU)	Catalytic subunit (CSU) or large subunit of acetolactate synthase (ALS)	Group B (inhibition of ALS), first gene in BCAA pathway
*ALS* (RSU)	Regulatory subunit (RSU) or small subunit of acetolactate synthase (ALS)	Group B (inhibition of ALS), first gene in BCAA pathway
*TD*	Threonine dehydratase (deaminase)	BCAA pathway
*KARI*	Ketol-acid reductoisomerase	BCAA pathway
*DHAD*	Dihydroxy-acid dehydratase	BCAA pathway
*BCAT-2*	Branched-chain-amino-acid aminotransferase (isoform 2 sequences are the most commonly available)	BCAA pathway
*IPMS*	2-Isopropylmalate synthase 1	BCAA pathway
*IPMI*	Isopropylmalate isomerase (dehydratase)	BCAA pathway
*IMDH*	3-Isopropylmalate dehydrogenase	BCAA pathway
*aroA*	5-enolpyruvylshikimate-3-phosphate (EPSP) synthase	Group G (inhibition of EPSP synthase)
*accA*	Acetyl-coA carboxylase subunit alpha	Group A (inhibition of ACCase)
*psbA*	Photosystem II protein D1	Group C (inhibition of photosynthesis PS II)

*The relevant herbicides are grouped based on mode of action following the Herbicide-Resistance Action Committee classification system (Beffa et al., 2019).*

To recover the full-length sequence of each gene from the genome data generated for each weed species, we first searched among the protein sequences predicted from the genome data, using the corresponding plant protein sequences as queries in BLASTp searches. To curate these query sequences, relevant plant protein sequences that are categorized as InterPro families, i.e., ALS CSU (IPR012846), ALS regulatory subunit (RSU; IPR004789), threonine dehydratase (TD; IPR005787), ketol-acid reductoisomerase (KARI; IPR013023), and branched-chain-amino-acid aminotransferase (BCAT-2; IPR005786), were downloaded from the InterPro database on October 15, 2020. For the other proteins not classified as InterPro families, the sequences were retrieved from UniProt and NCBI GenBank. The complete list of query sequences is shown in Supplementary Table 3. If full-length protein was not recovered this way, their coding sequences were searched among the assembled genome sequences using known protein sequences as query (tBLASTn). If no full-length gene or protein sequence were recovered in these two steps, a gene-targeted assembly approach was adopted to recover the full-length coding sequence using MegaGTA v0.1-alpha (Li et al., 2017). Briefly, for each weed species, a multiple sequence alignment and the associated Hidden Markov model profile (i.e., the alignment profile) for the target gene were first created using homologous sequences from taxa within the same Order (Supplementary Table 4), which were sourced from InterPro release 80.0 and the associated databases within. The alignment and the associated profile were then used to recover the target protein-coding sequence in full length. Given that conservation of gene functions are manifested in the coded protein sequences, we used the predicted protein sequences from each genome in subsequent analysis.

### Validation of ALS CSU From African Boxthorn Using Polymerase Chain Reaction

As a proof of concept of our targeted approach using whole-genome data, we validated the ALS CSU coding sequence from African boxthorn using a PCR-based primer-walking approach. Primers (Supplementary Table 5) were designed based on highly conserved regions in the middle of ALS_CSU coding sequences. During the first phase, a PCR reaction was performed on the genomic DNA using degenerate primers, A1F and A1R, from which the amplified product together with the primers B1F and B1R were used in a second PCR reaction. The final amplified product was verified using agarose (1% w/v) gel electrophoresis, extracted using the QIAquick gel extraction kit (Qiagen, United States) following the manufacturer’s procedure, and sequenced using the Sanger method at the Australian Genome Research Facility (Brisbane, Australia). This sequence was then used to design primers targeting up-/down-stream regions, as part of the primer-walking strategy; these primers include those targeting sequence coding for the N-terminal (C1R, C2R) and C-terminal (D1F) protein regions, including degenerate primers (C1F, D1R) designed based on other similar ALS_CSU sequences from other closely related plants. During the second phase, the sequence coding for the N-terminal region was identified by performing a PCR reaction on the genomic DNA using primers C1F and C1R, from which the amplified product together with primers C1F and C2R were used in a second PCR reaction. An independent PCR reaction was performed on the genomic DNA using primers D1F and D1R to verify the sequence coding for the C-terminal region. The sequence coding for the mitochondrial transit peptide is highly variable, and thus cannot be determined using this approach.

Each PCR reaction mix (50 μL) contained 1 × Thermopol^®^ buffer, 0.2 mM dNTPs, forward primer (1 μM), reverse primer (1 μM), template DNA (∼1000 ng), and *Taq* DNA polymerase (3 U). If genomic DNA was the template, 4 μL (∼30 ng) of the purified DNA was used. For each PCR reaction, initial denaturation (95°C, 1 min) was followed by 35 cycles of denaturation (95°C, 20 s), annealing [primer-specific temperature (Supplementary Table 5), 30 s], and elongation (70°C, 45 s; 1 min for the 35th elongation step). The reaction was kept at 10°C prior to subsequent analysis.

### Assessment of Conserved Amino Acids and Target Sites for ALS and EPSP Synthase

For each of ALS RSU, ALS CSU, and EPSP synthase, we compared protein sequences from the seven weed species against the structurally resolved protein sequence of a related species as reference. For ALS, we used the *At*RSU (UniProt Q93YZ7; PDB 6VZ8) and *At*CSU (UniProt P17597; PDB 1YBH) from *Arabidopsis thaliana*. For EPSP synthase, the protein from *Escherichia coli* (UniProt P0A6D3; PDB 1G6S) was used; no protein structures for plant EPSP synthase have been resolved. For each set of protein sequences, multiple sequence alignment was performed using Cluster Omega (Sievers et al., 2011). To assess interaction of a protein with other molecules, we used PoseView^2^ (Stierand et al., 2006) to generate two-dimensional representations of the molecular complex formed by a protein interacting with substrate, cofactor, and herbicide molecules.

### Inference of Phylogenetic Tree

For each gene, the query sequences used in the initial BLASTp searches (Supplementary Table 3) and the gene sequences recovered from the weed species were combined to reconstruct a homologous protein-sequence set; this yielded 12 homologous sets. For each set, multiple sequence alignment was performed using MAFFT v7.471 (Katoh and Standley, 2013) in L-INS-i mode. For the analysis of the 18S rRNA sequences, multiple sequence alignment was performed using MUSCLE v3.8.31 (Edgar, 2004). Aligned sequence positions that are not parsimoniously informative were removed with trimAl v1.4.rev15 (Capella-Gutiérrez et al., 2009) using the *automated1* setting. Using the processed alignment as input, a maximum-likelihood phylogenetic tree was inferred using IQ-TREE v2.0.3 (Nguyen et al., 2015) with the most appropriate substitution model chosen using ModelFinder (Kalyaanamoorthy et al., 2017), and node support assessed using an ultrafast bootstrap approximation (Hoang et al., 2018) based on 2,000 sample replicates.

## Results and Discussion

### Genome Data From Seven Weed Species

We generated new genome data from seven invasive weed species in Australia to guide our identification of gene targets for common herbicides, focusing on glyphosate and ALS inhibitors. Supplementary Table 2 shows the statistics for each assembled draft genome and the associated number of predicted protein-coding genes. We further assessed the completeness of these genome data based on the recovery of core conserved single-copy genes in Viridiplantae (i.e., the group encompassing all plants and green algae) using the standard benchmark tool for genome or gene-repertoire completeness, BUSCO (Waterhouse et al., 2018); in this context, a high recovery of these genes (e.g., >90%) indicates that the genome data are near complete.

All draft genomes are highly fragmented with a high number of scaffolds (>80,000) and short N50 lengths (<21 Kbp). For instance, the fragmented genome assembly for alligator weed (1,160,435 scaffolds, N50 length 3.30 Kbp) is likely due to its large genome size, i.e., only 1.75 Gbp was recovered in the assembly compared to the estimated size of 4.97 Gbp; this is also confirmed by the low (47.0%) genome completeness based on BUSCO analysis. In contrast, the most contiguous assembly is the rubber vine genome (82,815 scaffolds, N50 length 20.26 Kbp), with the estimated genome size of 0.48 Gbp (0.38 Gbp recovered in the assembly) and BUSCO recovery at 88.5%. Based on the common measures of contiguity of assembled genome sequences, the quality of these assemblies is insufficient for a comprehensive comparative genomic analysis, as the predicted protein-coding genes are likely fragmented, and the gene repertoire incomplete. As these sequence data were generated *de novo* with no good reference available, ploidy number and genome size cannot be readily identified from the data without further experiments. However, these data remain suitable for identifying specific genes in each weed species that are targeted by glyphosate and ALS inhibitors; these genes are expected to be present in all plant genomes. Moreover, not considering the assembled genome of alligator weed, all the other six assembled genomes have an average BUSCO completeness of 79.5% (between 62.9% and 90.6%; Supplementary Table 2), suggesting that most protein-coding genes encoded in these weed genomes are present in these albeit fragmented, *de novo* assembled sequences.

Given the fragmented genome assemblies, we took additional precaution to ensure that the data were truly representative of each weed species. Our comparison of 18S rRNA sequences from the seven genomes against those from other plant species revealed robust groupings (node support > 90%) of each weed species with their closely related species or genus, within the expected Order. For instance, fireweed and parthenium grouped within Asterales, while water hyacinth, the only monocot among the seven weed species, grouped within Order Commelinales as expected, close to other monocot Orders such as Asparagales (Supplementary Figure 2). This relationship was also supported at the protein level by the individual trees of two subunits of the ALS protein, i.e., the catalytic subunit (CSU; Supplementary Figure 3), and the regulatory subunit (RSU; Supplementary Figure 4). These results provide first-line molecular evidence for the identity of the seven weed species from which the genome data were generated.

### Genome-Guided Identification of Genes Targeted by Herbicides

**FIGURE 2:**
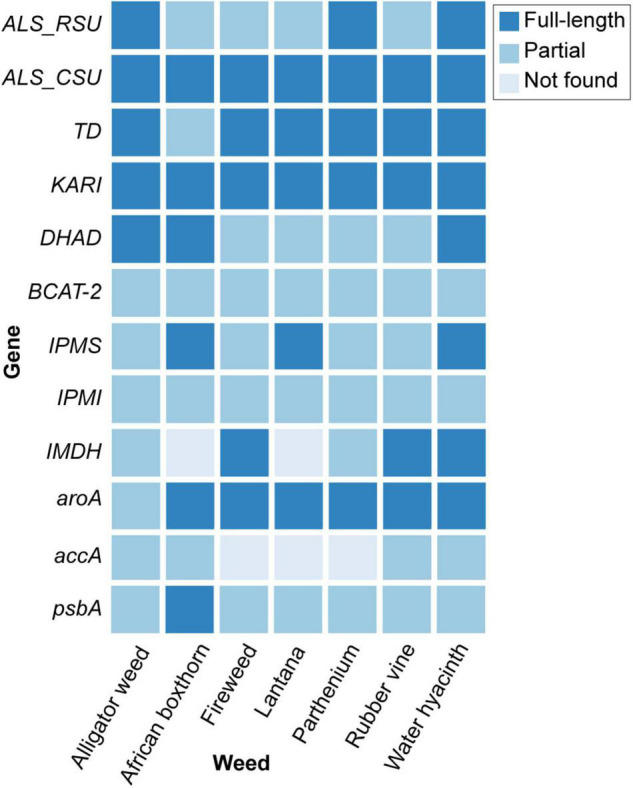
The 12 key genes identified in the genomes of seven weed species.

From the genome data, we adopted an integrated approach (Materials and Methods) to identify, in each weed species, 12 key genes that are current or potential targets for common herbicides, particularly glyphosate and ALS inhibitors, including seven involved in the BCAA pathway (Table 2 and Figure 2; see Section “Materials and Methods”). The identified gene and protein sequences from each genome are available as Supplementary Data Sheet 1. The *ALS* gene is a current target for commercial herbicides, and for this we focus on the catalytic subunit (CSU) of ALS. However, other potential binding sites can be found in the regulatory subunits (RSU) (Lonhienne et al., 2020). Inhibitors of other enzymes in the BCAA pathway, e.g., ketol-acid reductoisomerase (KARI) (Biou et al., 1997) and dehydroacid dehydratase (DHAD) (Yan et al., 2018) are also known to exhibit good herbicidal activity, thus detailed knowledge of the genes encoding these enzymes or regulators is useful for future efforts in herbicide discovery.

All 12 genes were recovered in the seven genomes, except for the *accA* (recovered in four) and *IMDH* (recovered in five) (Figure 2). This observation was likely due to the fragmented nature of the draft genome sequences, which also affects genome quality and completeness (Supplementary Table 2), not because of loss of these genes in these genomes. Recovery of most of these genes from the draft genomes demonstrates the utility of our high-throughput genome-sequencing approach for identifying gene targets in leiu of the more-tedious targeted gene sequencing approach (e.g., *via* amplification of specific gene regions).

#### Catalytic Subunit of Acetolactate Synthase

The CSU of ALS contains the target site for the largest number of commercial herbicides (Garcia et al., 2017). The holoenzyme of ALS also contains an RSU that binds to the CSU (Lonhienne et al., 2020). The RSU upregulates the activity of the enzyme by ∼6-fold. This enhancement of the activity can be offset in the presence of the BCAAs (Lonhienne et al., 2020). The cryo-EM structure of the complex suggests that the presence of the RSU could affect the affinity of herbicide binding to the CSU, and could therefore be of relevance in explaining herbicidal activity for specific species.

We identified the complete sequences of the CSUs for all seven weed species (Figure 2); the coding sequence for the CSU in African boxthorn was further validated using PCR in a primer-walking approach (see Section “Materials and Methods”). The CSU is the target of five commercial herbicide families (see examples below) that bind to the catalytic pocket, thus blocking active site access to substrates (i.e., pyruvate and 2-ketobutyrate) (McCourt and Duggleby, 2006).

**FIGURE 3:**
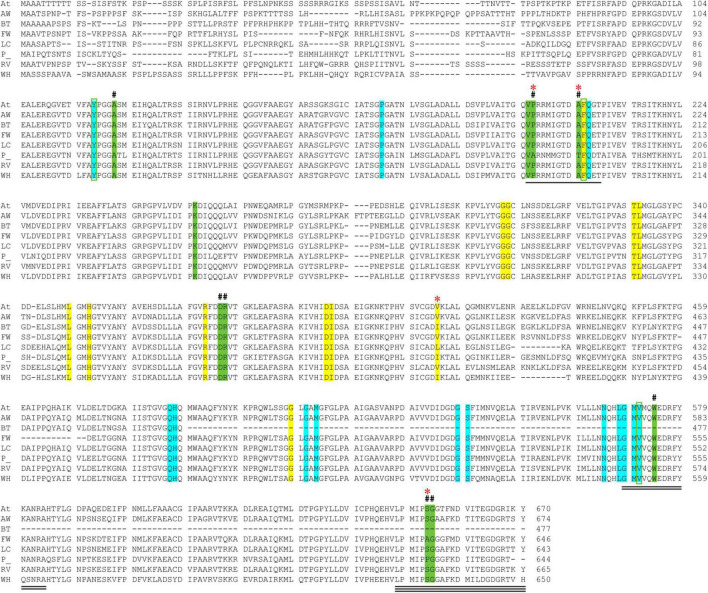
Alignment of *At*CSU and the CSU sequences from the seven weed species. The catalytic loop, the mobile loop, and the C-terminal arm are underlined (1, 2, and 3 lines, respectively). The residues that interact with ThDP (highlighted in cyan), FAD (yellow), and the herbicide chlorimuron ethyl (CE; green, including three positions bounded in green boxes) are highlighted. A hash (#) above the alignment represents the mutation sites responsible for resistance to herbicides. A red asterisk (*) above the alignment designates residues that are not fully conserved across the eight sequences. Legend: *A. thaliana* (At), alligator weed (AW), African boxthorn (BT), fireweed (FW), lantana (LC), parthenium (P), rubber vine (RV), and water hyacinth (WH).

The CSU sequences were analyzed for the conservation of residues that are essential for the binding of herbicides and for ALS activity (i.e., the catalytic loop and the binding sites for the cofactors ThDP and FAD) (Figure 3). The *Arabidopsis thaliana* CSU (*At*CSU) was chosen as the model to analyze relevant structural features because it is the only plant CSU for which experimentally verified structural information (i.e., crystal structures) are available (McCourt et al., 2006; Garcia et al., 2017).

**FIGURE 4:**
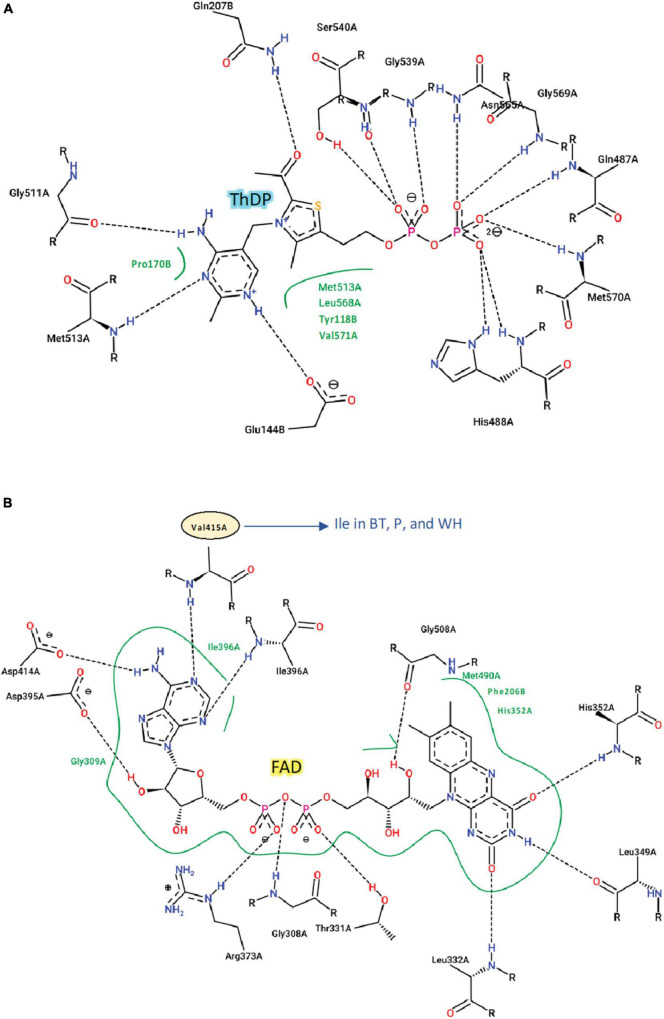
Interactions of ThDP, FAD with *At*CSU. Two-dimensional representation of the interactions of **(A)** ThDP and **(B)** FAD with *At*CSU. Residues labeled in black and green form hydrogen bonds and hydrophobic interactions, respectively. BT, African boxthorn; P, parthenium; WH, water hyacinth.

The catalytic loop [also known as the Q-loop (Lonhienne et al., 2018)] is highly conserved across all species (Figure 3), with F206 and Q207 (based on numbering of *At*CSU; this numbering is used hereinafter) being invariant. These residues directly interact with the substrate and have critical roles in catalysis (Lonhienne et al., 2017). Residues that interact with ThDP are also fully conserved (Figure 4A), and those that interact with FAD are highly conserved, with only one residue that is variable; Val415 in *At*CSU is replaced by isoleucine in African boxthorn, partenium, and water hyacinth (Figure 4B). Presumably, this modification has no effect on the binding of FAD as the interaction involves only the nitrogen atom of the backbone amide group of Val415. In general, the high conservation of all these sites strongly suggests that all the weed CSU sequences assembled and identified here are fully functional (Figure 2).

**FIGURE 5:**
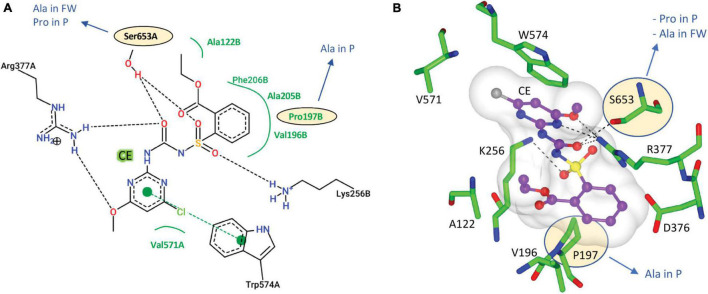
Interactions of CE with *At*CSU. **(A)** Two-dimensional representation of CE interacting with *At*CSU. Residues labeled in black and green form hydrogen bonds and hydrophobic interactions, respectively. **(B)** Three-dimensional representation of CE interacting with *At*CSU (PDB ID 1YBH). The residues with observed variations in the seven weeds are circled. FW, fireweed; P, parthenium.

The CSU is the target for 58 commercial herbicides (Garcia et al., 2017). These herbicides fall into five different chemical classes, sulfonylureas (SUs), imidazolinones (IMIs), triazolopyrimidines (TPs), pyrimidinylbenzoates (PYBs), and the sulfonylamino-carbonyl-triazolinones (SCTs) (Garcia et al., 2017). Since the introduction of these herbicides in the 1980s, eight mutation sites that confer resistance have been reported (Heap, 2022). All mutations are in the catalytic pocket in regions that are in contact with the inhibitor (Figure 3). We chose chlorimuron ethyl (CE, a sulfonylurea) for investigation, as it is one of the most common commercial herbicides utilized in the field, and a three-dimensional structure has been solved in complex with *At*CSU (PDB identifier: 1YBH). The sequence alignment of the CSUs shows that amongst the 13 residues that interact with CE, which includes the eight residues for which mutations have been reported (Heap, 2022), three are variable (Figure 3). The first, corresponding to Pro197 in *At*, has been replaced by alanine in parthenium (Figure 5A). The second, corresponding to Ser653 in *At*, has been replaced by proline and alanine, in parthenium and fireweed, respectively (Figure 5B). The third corresponds to Ala205, a residue which to date has not been reported as a resistance mutation site. These results suggest that in all seven weed species, residues involved in herbicide binding are generally highly conserved. For five species (alligator weed, African boxthorn, lantana, rubber vine, and water hyacinth), there is no variation suggesting that the efficiency of herbicides should not be affected should alternate weed resistance mechanisms prevent the herbicide reaching the binding site. The CSUs coded in the genomes of fireweed and parthenium vary at S653 (Figure 5B), which is one of the major resistance sites that appear in weeds subjected to ALS-inhibiting herbicides (Heap, 2022). The S653 mutation affects the efficacy of the herbicides of the PYBs, IMIs, and SCTs families (Heap, 2022), thus these herbicides are likely ineffective for controlling parthenium and fireweed. The additional variation in parthenium, where P197 replaced by an alanine residue is also a site where mutation provides resistance, suggests that the ALS-inhibiting herbicides may not be suitable for application against parthenium.

#### Regulatory Subunit of Acetolactate Synthase

**FIGURE 6:**
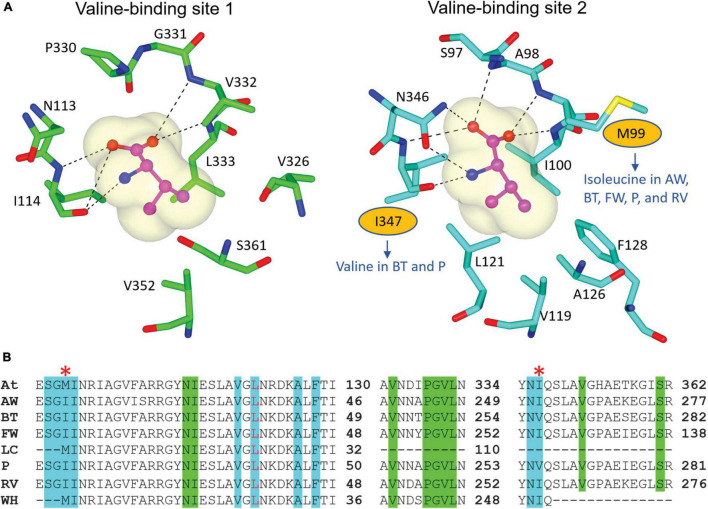
Residues involved in BCAA binding in the RSU. **(A)** Valine-binding sites in *At*RSU (PDB ID 6VZ8). The residues where there are variations in the weed sequences are circled. **(B)** Alignment of the RSU sequences of the seven weeds and *At*RSU. The residues that interact with valine in binding site 1 and 2 are highlighted in green and cyan, respectively. A red asterisk (*) above the alignment designates residues that are not fully conserved across the eight sequences. At, *A. thaliana*; AW, alligator weed; BT, African boxthorn; FW, fireweed; LC, lantana; P, parthenium; RV, rubber vine; WH, water hyacinth.

We recovered full-length RSU sequences from the genomes of alligator weed, parthenium, and water hyacinth, and partial sequences from those of the other four species (Figure 2). The RSU plays an important role in regulating the activity of ALS. Binding of RSU to the CSU increases ALS activity, but also allows feedback inhibition by the BCCAs (Lonhienne et al., 2020). All recovered RSU sequences have the regions implicated in BCAA binding. There are two different binding sites for the BCAAs in the plant enzyme, both can bind valine, but their specificity for the individual BCAAs is likely to be different (Lonhienne et al., 2020). The alignment of eight RSU sequences (from seven weeds and *A. thaliana*) shows complete conservation of the residues at binding site 1 (Figure 6A), and two variable residues (M99 and I347) at binding site 2 (Figure 6B). Presumably, the variations at binding site 2 do not affect its functionality, because these residues interact with valine through backbone carbonyl and amide groups. The high degree of conservation across all seven weed species suggests that these sites are attractive for the continued development of novel inhibitors with potential as new herbicide leads.

#### EPSP Synthase, the Target of Glyphosate

**FIGURE 7:**
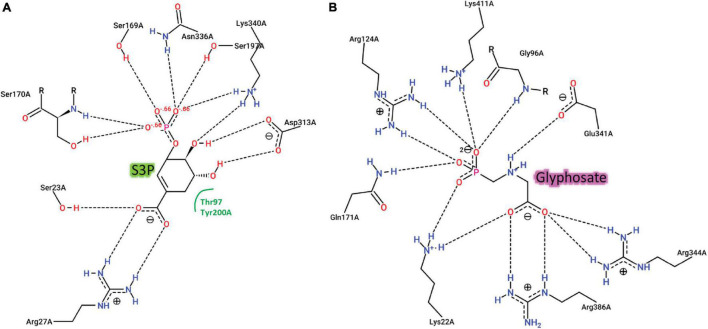
Two-dimensional representations of the interactions of *E. coli* EPSPS with **(A)** the substrate, S3P and **(B)** the herbicide, glyphosate.

**FIGURE 8:**
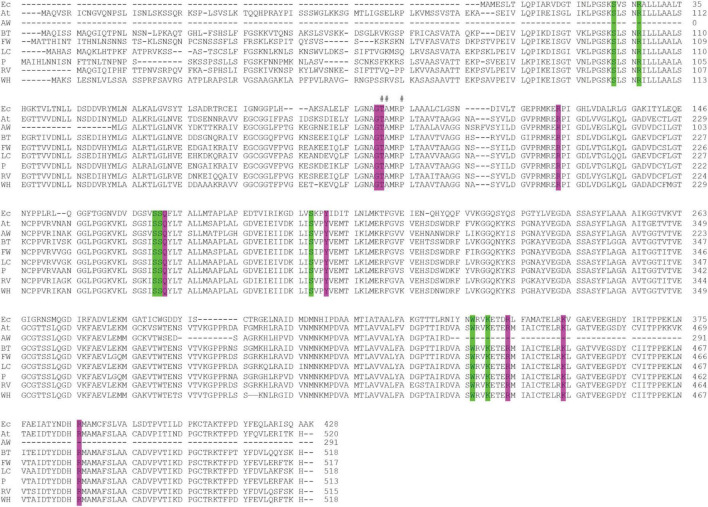
Alignment of the EPSP synthase sequences of the seven weeds, *A. thaliana* and *E. coli*. The residues that interact with the S3P and glyphosate are highlighted in green and purple, respectively. A hash (#) above the alignment represents the mutation sites responsible for resistance to glyphosate (Heap, 2022). Ec, *E. coli*; At, *A. thaliana*; AW, alligator weed; BT, African boxthorn; FW, fireweed; LC, lantana; P, parthenium; RV, rubber vine; WH, water hyacinth.

We recovered full-length protein sequences of EPSP synthase in all genomes except for the genome of alligator weed, in which a partial sequence was recovered (*aroA* in Figure 2). EPSP synthase, an enzyme in the shikimate pathway involved in the synthesis of aromatic amino acids, is the target for glyphosate (Funke et al., 2006). EPSP synthase catalyzes the transfer of the enolpyruvyl moiety of phosphoenolpyruvate (PEP) to shikimate-3-phosphate (S3P), generating 5-enolpyruvyl-shikimate-3-phosphate (EPSP). Glyphosate competes with PEP, mimicking an intermediate state of the ternary enzyme-substrates complex (Funke et al., 2006). Currently, no crystal structure of a plant EPSP synthase is available, although the structure of *Escherichia coli* EPSP synthase in complex with S3P and glyphosate [PDB identifier: 1G6S (Funke et al., 2006)] has been solved, which identifies residues that interact with S3P and glyphosate (Figure 7). The *E. coli* EPSP synthase shared high sequence similarity with its counterpart protein in plants (Figure 8), thus presenting an excellent model for comparison. An alignment of the EPSP synthase sequences from *At* and the seven weed species shows that all residues involved in the binding of S3P and glyphosate are highly conserved (Figure 8). Three mutation sites that confer resistance have been reported (Heap, 2022). These three residues are also totally conserved (Figure 8). These results demonstrate that glyphosate should bind potently to the EPSP synthase from these weeds.

### Vertical Inheritance of Genes Targeted by Herbicides

For each of the 12 proteins, we further assessed their evolutionary relationship among plant taxa. Similar to what we observed for the CSU (Supplementary Figure 3) and RSU (Supplementary Figure 4) of ALS, each phylogenetic tree of the seven proteins implicated in the BCAA pathway (TD, KARI, DHAD, BCAT-2, IPMS, IPMI, and IMDH; Supplementary Figures 5–11) suggests vertical inheritance of the genes, in which all seven weed species are positioned within monophyletic clades of their corresponding Orders among seed plants (spermatophytes) (The Angiosperm Phylogeny Group, 2009). We observed a similar trend in the protein trees for AroA (Supplementary Figure 12) and accA (Supplementary Figure 13), but not in the tree of psbA (Supplementary Figure 14), in which the various Orders are not grouped in monophyletic clades. In contrast to the other 11 genes that are encoded in the nuclear genome of plants, gene *psbA* is encoded in the chloroplast genome. Our tree (Supplementary Figure 14) supports the notion that psbA is not suitable as a phylogenetic marker for plants, in contrast to the established chloroplast marker gene *rbcL* (Hollingsworth et al., 2009).

## Concluding Remarks

Our results demonstrate the use of whole-genome sequencing data from invasive weeds to quickly screen for potential gene and protein targets for herbicides, without the need for high-quality assembled genomes that are commonly cost-prohibitive in plants mainly because of their large genome sizes and the complication of polyploidy. Based on comparative sequence analysis, we identified conserved amino acids and target sites for the widely used herbicides (e.g., ALS inhibitors and glyphosate), and that most of these protein targets are evolutionarily conserved in plants through vertical inheritance. Compared to the other examined weed species, fireweed and parthenium, for which mutations were identified at the target sites of ALS protein, are likely more resistant to ALS-inhibiting and other herbicides. Although this hypothesis remains to be tested experimentally, given the high sequence conservation we observed among the seven phylogenetically diverse species, the proteins we identified present excellent models for further research without the need for expressing and purifying the proteins individually.

While we focused on targets of two major herbicides in this study, our approach and the data we generated can be readily applied to identify targets of *de novo* herbicides including novel leads that are under development, and other proteins relevant to herbicide tolerance (Werck-Reichhart et al., 2000; Dimaano and Iwakami, 2021). These data present a useful resource of sequence information that can be exploited for future efforts in herbicide discovery and development. They serve as a potential guide for selecting or designing herbicides, particularly in sustaining crop improvement.

## Data Availability Statement

The datasets presented in this study can be found in online repositories. The names of the repository/repositories and accession number(s) can be found below: https://www.ncbi.nlm.nih.gov/bioproject/, PRJEB47218. All assembled genomes, and the predicted gene and protein sequences are available at https://doi.org/10.48610/c22c10c.

## Author Contributions

TL, GS, LG, and CXC conceived the study and designed the research. SS, YC, and KD performed bioinformatic analysis of genome data, gene-target identification, and phylogenetic analyses. TL and YL performed computational analysis of protein-sequence conservation. C-EM and TL contributed to collection of biological material and data generation for this study. SS and TL prepared the first draft of the manuscript. C-EM, CW, GS, GW, LG, and CXC contributed to writing and iterative revisions of the manuscript. All authors reviewed and approved the final manuscript.

## Conflict of Interest

The authors declare that the research was conducted in the absence of any commercial or financial relationships that could be construed as a potential conflict of interest.

## Publisher’s Note

All claims expressed in this article are solely those of the authors and do not necessarily represent those of their affiliated organizations, or those of the publisher, the editors and the reviewers. Any product that may be evaluated in this article, or claim that may be made by its manufacturer, is not guaranteed or endorsed by the publisher.

## Footnotes

1
https://keyserver.lucidcentral.org/weeds

2
https://www.biosolveit.de/free-to-academics/
