# Supplementary material for: Genome-Guided Analysis of Seven Weed Species Reveals Conserved Sequence and Structural Features of Key Gene Targets for Herbicide Development
Source: Front Plant Sci. 2022 Jun 29;13:909073. doi: 10.3389/fpls.2022.909073 (PMC9277346; doi:10.3389/fpls.2022.909073)
Supplement: Supplementary file 1 [file Image_1.PDF]

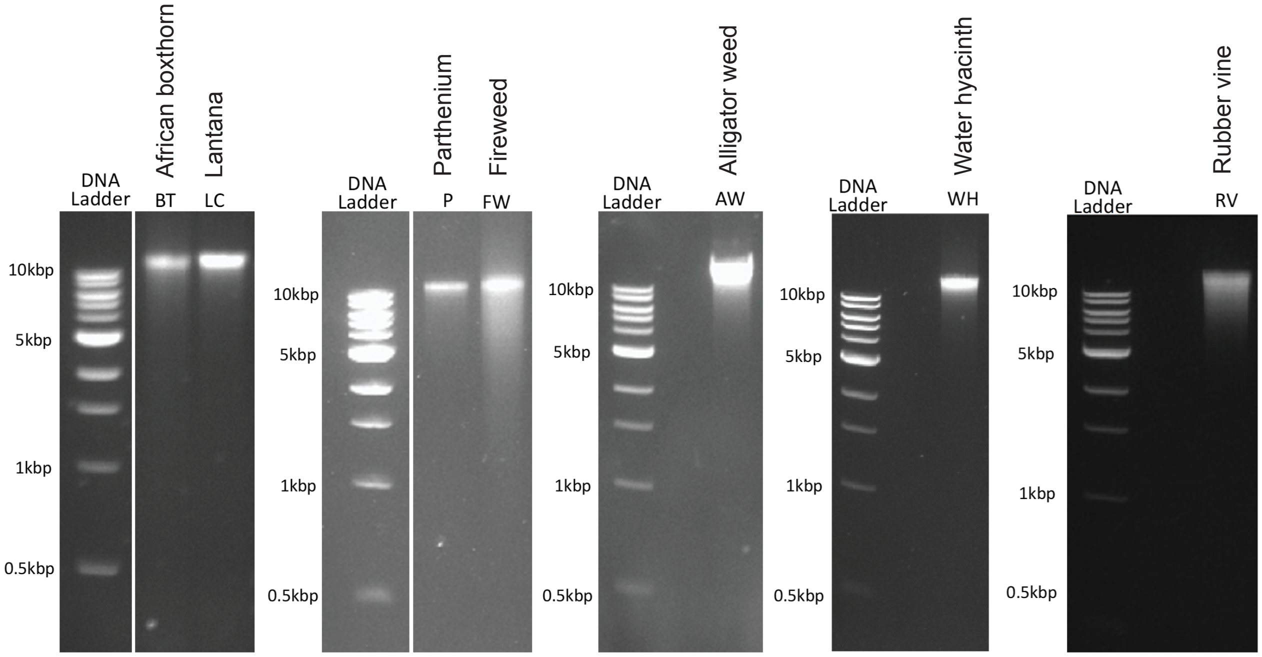

Supplementary Figure 1. Agarose gel electrophoresis of the purified genomic DNA for the seven weed species.
