# Supplementary material for: Genome-Guided Analysis of Seven Weed Species Reveals Conserved Sequence and Structural Features of Key Gene Targets for Herbicide Development
Source: Front Plant Sci. 2022 Jun 29;13:909073. doi: 10.3389/fpls.2022.909073 (PMC9277346; doi:10.3389/fpls.2022.909073)
Supplement: Supplementary file 2 [file Image_2.PDF]

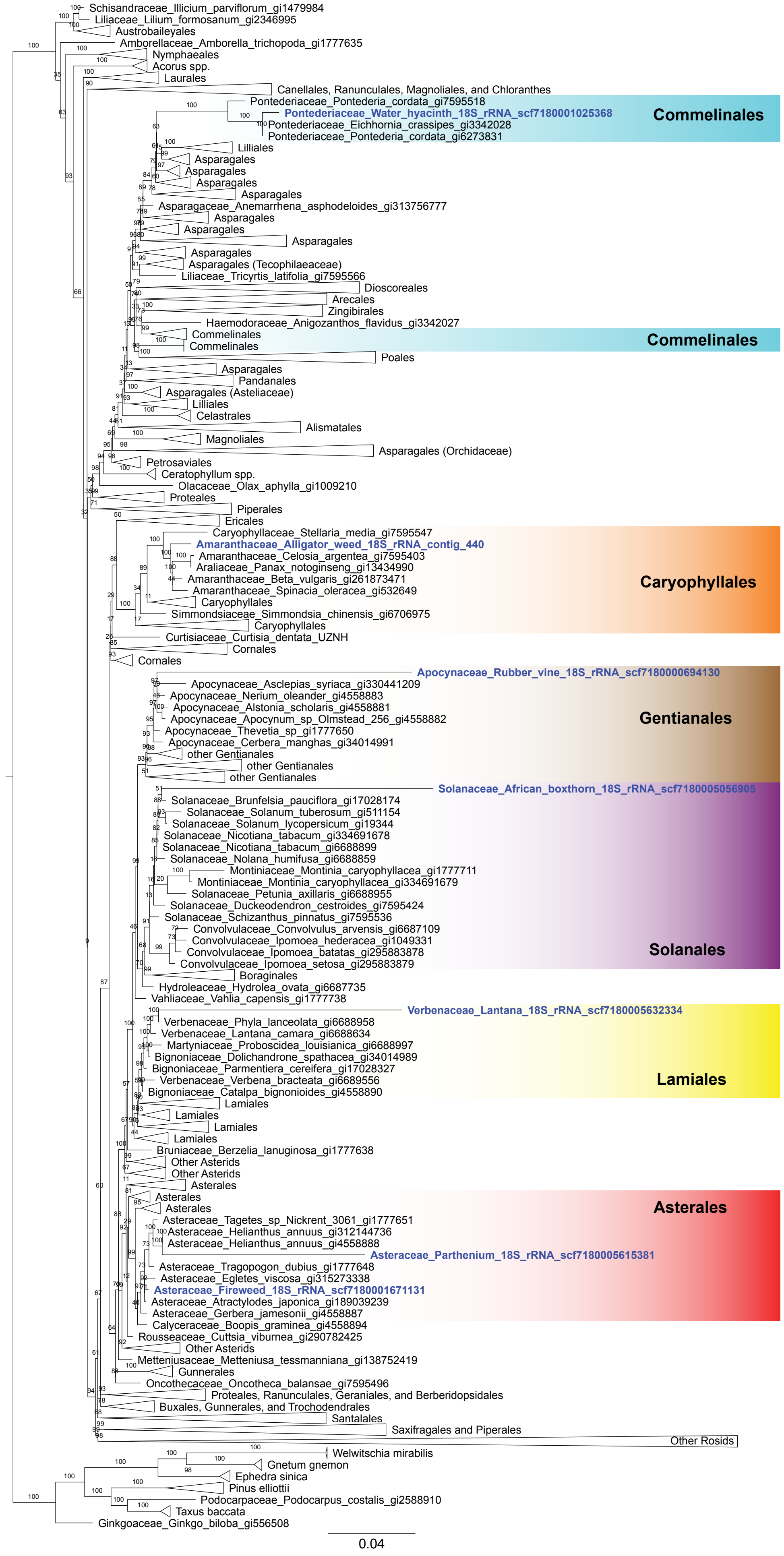

Supplementary Figure 2. Phylogenetic tree of plant taxa including the seven weed species used in this study, based on 18S rRNA sequences. The Order to which each species belongs is highlighted.
