# Supplementary figures and images for: Genome-Guided Analysis of Seven Weed Species Reveals Conserved Sequence and Structural Features of Key Gene Targets for Herbicide Development
Source: Front Plant Sci. 2022 Jun 29;13:909073. doi: 10.3389/fpls.2022.909073 (PMC9277346; doi:10.3389/fpls.2022.909073)

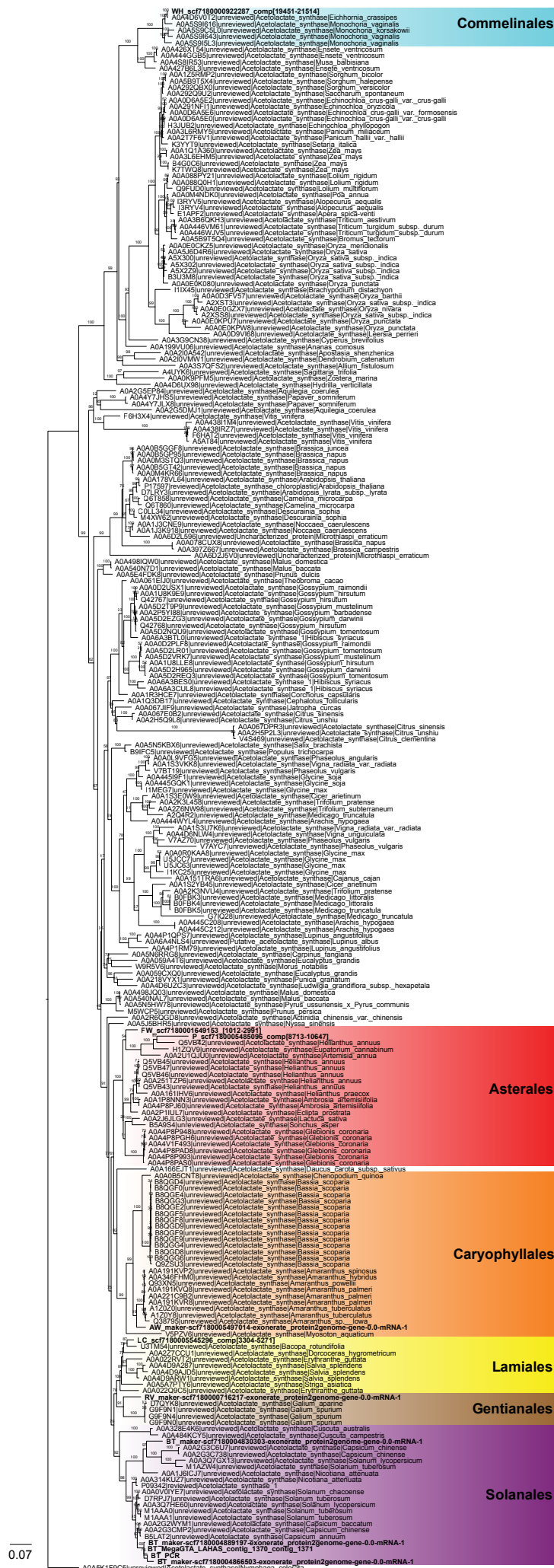

Supplementary Figure 3. Phylogenetic tree of plant taxa based on ALS CSU protein sequences.

Supplement: Supplementary file 3 [file Image_3.PDF]

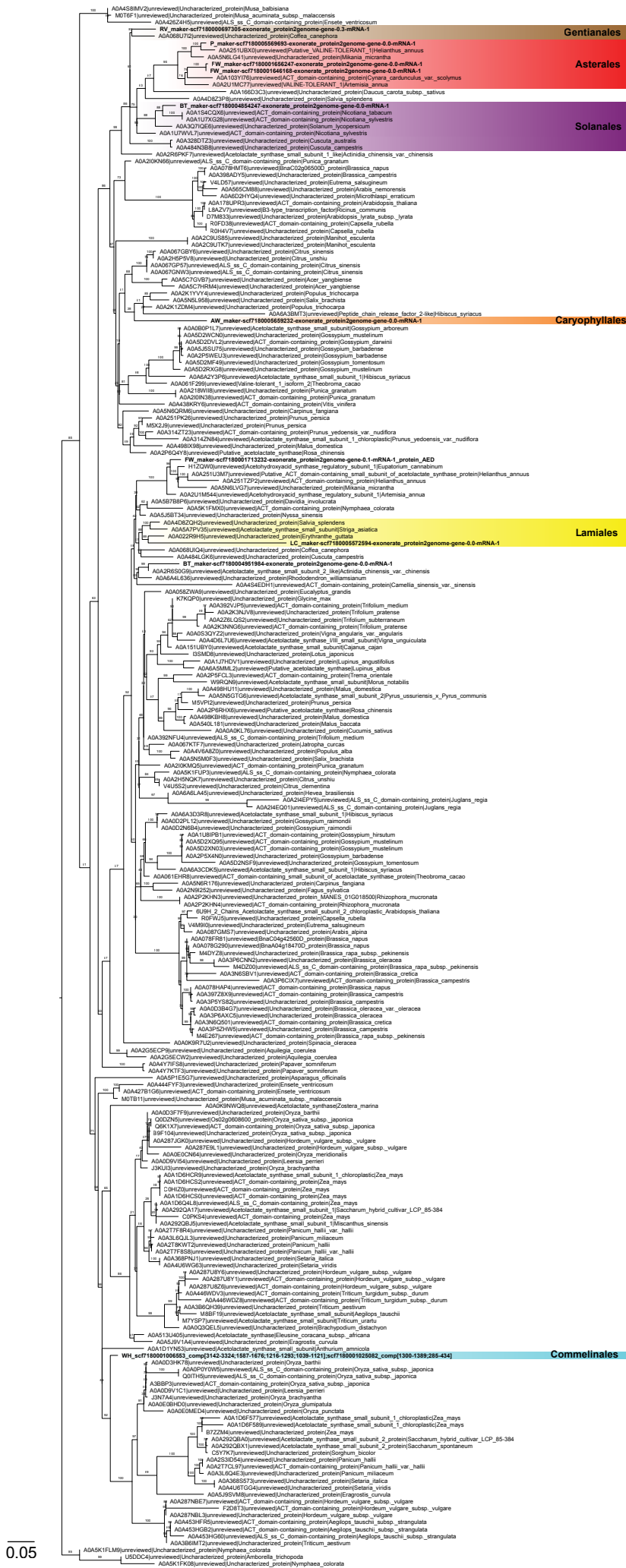

Supplementary Figure 4. Phylogenetic tree of plant taxa based on ALS RSU protein sequences.

Supplement: Supplementary file 4 [file Image_4.PDF]

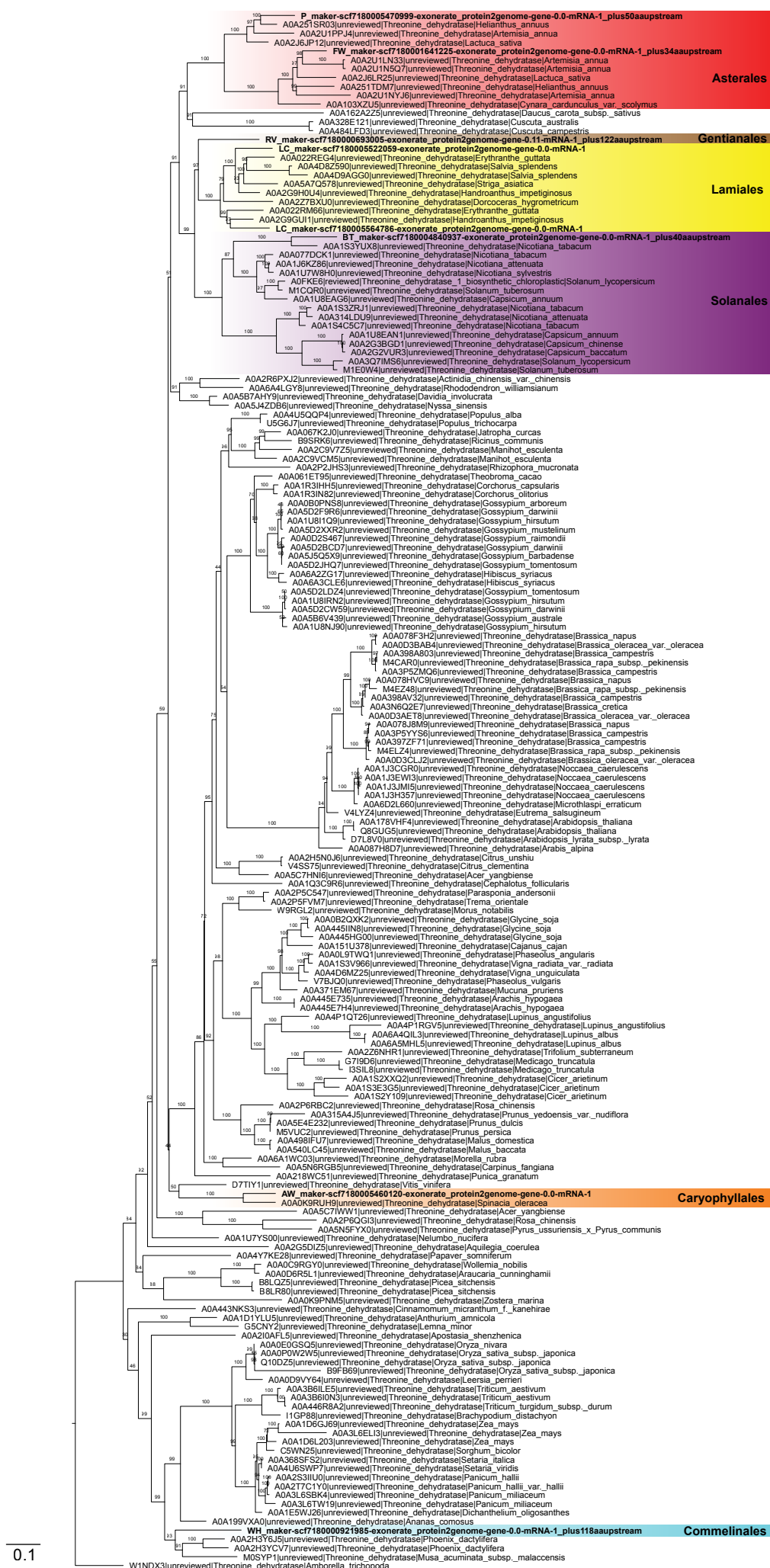

Supplement: Supplementary file 5 [file Image_5.PDF]

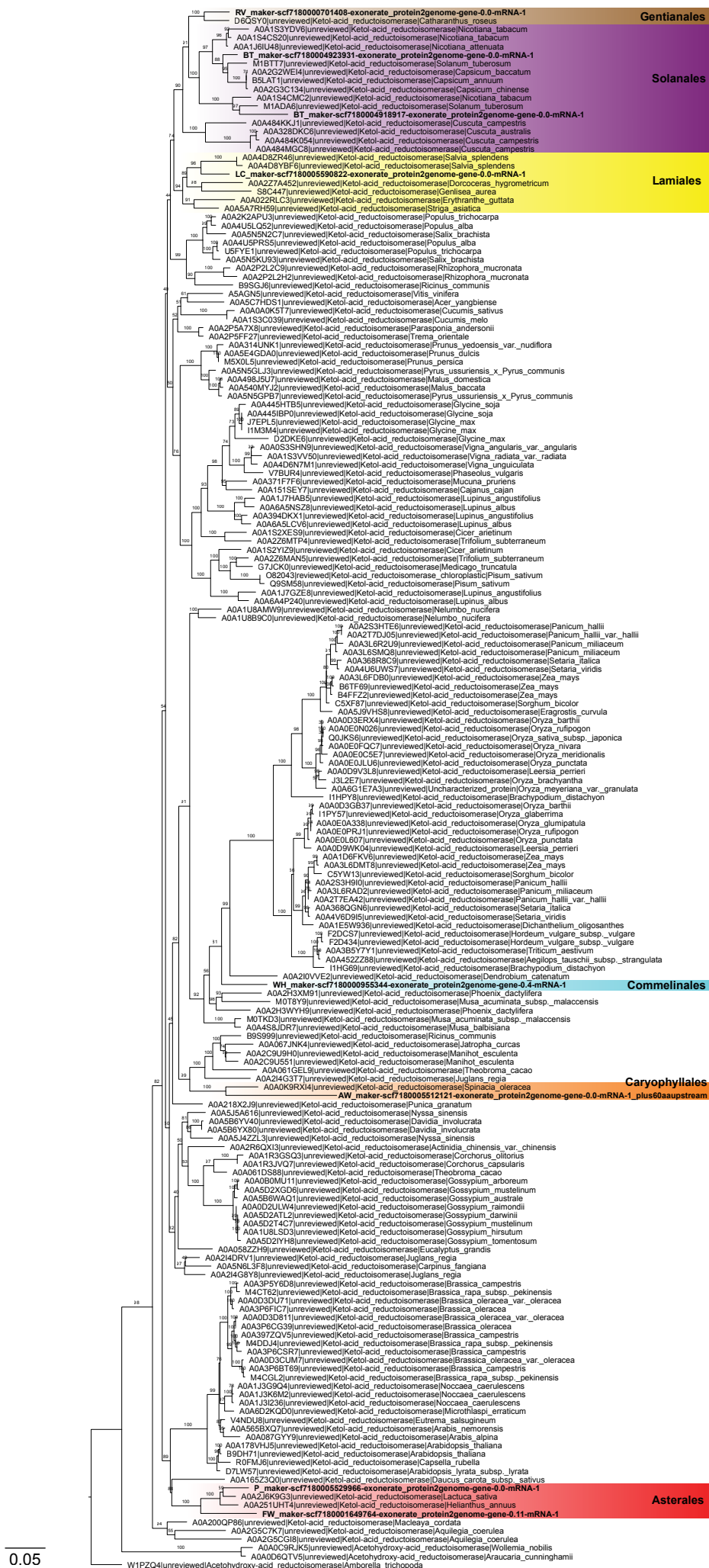

Supplementary Figure 6. Phylogenetic tree of plant taxa based on KARI protein sequences.

Supplement: Supplementary file 6 [file Image_6.PDF]

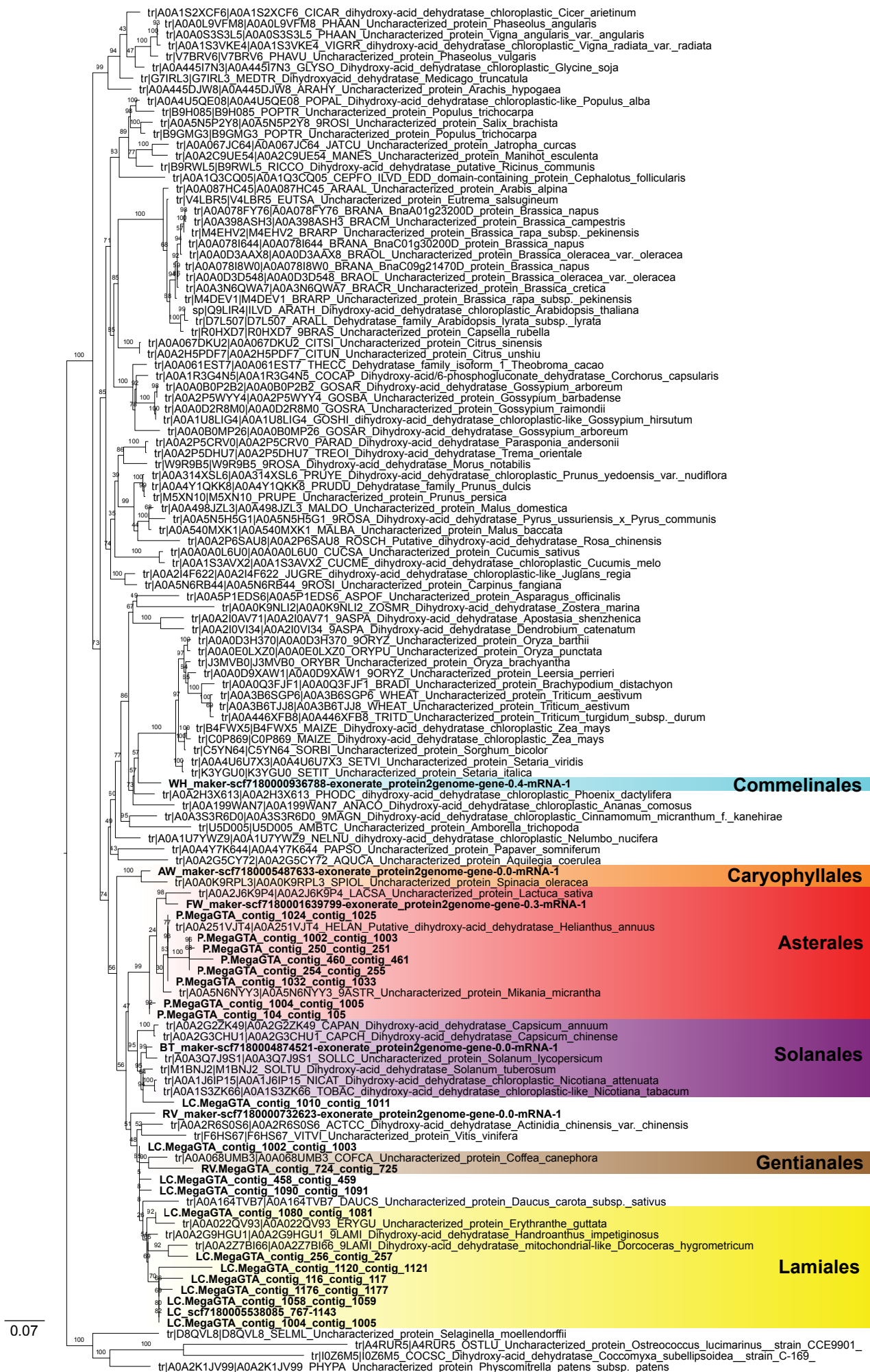

Supplementary Figure 7. Phylogenetic tree of plant taxa based on DHAD protein sequences.

Supplement: Supplementary file 7 [file Image_7.PDF]

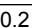

**Supplementary Figure 8.** Phylogenetic tree of plant taxa based on BCAT-2 protein sequences.

Supplement: Supplementary file 8 [file Image_8.PDF]

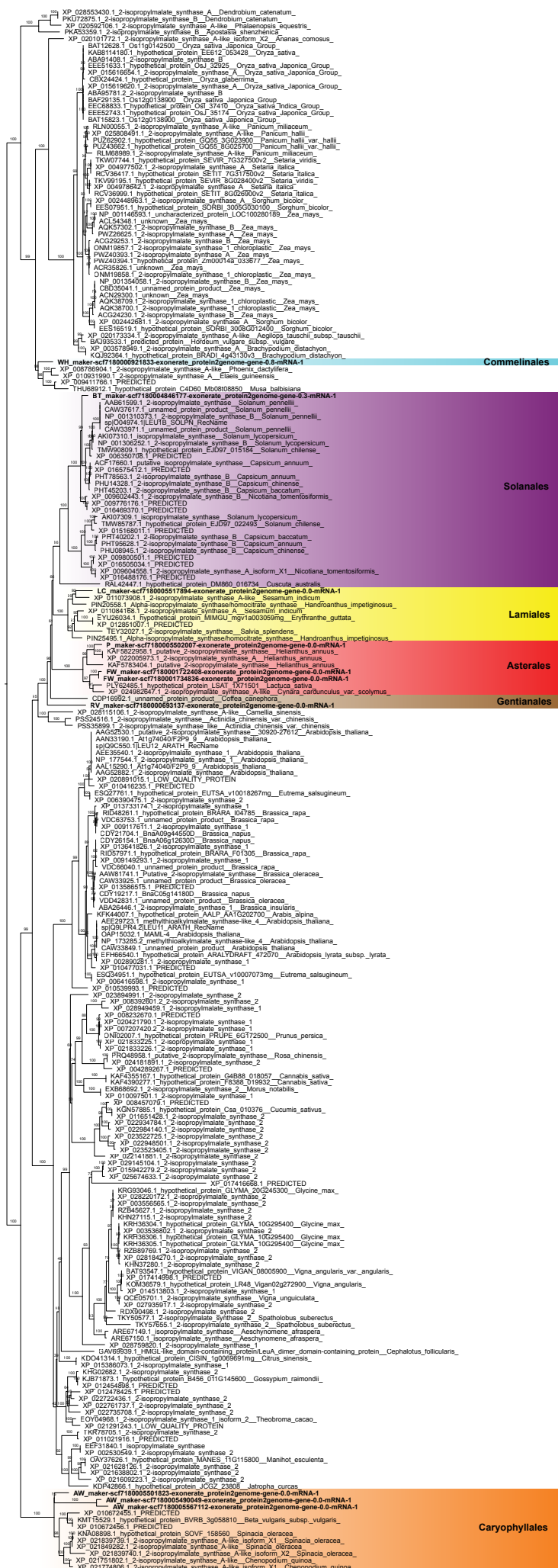

Supplementary Figure 9. Phylogenetic tree of plant taxa based on IPMS protein sequences.

Supplement: Supplementary file 9 [file Image_9.PDF]

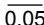

**Supplementary Figure 10.** Phylogenetic tree of plant taxa based on IPMI protein sequences.

Supplement: Supplementary file 10 [file Image_10.PDF]

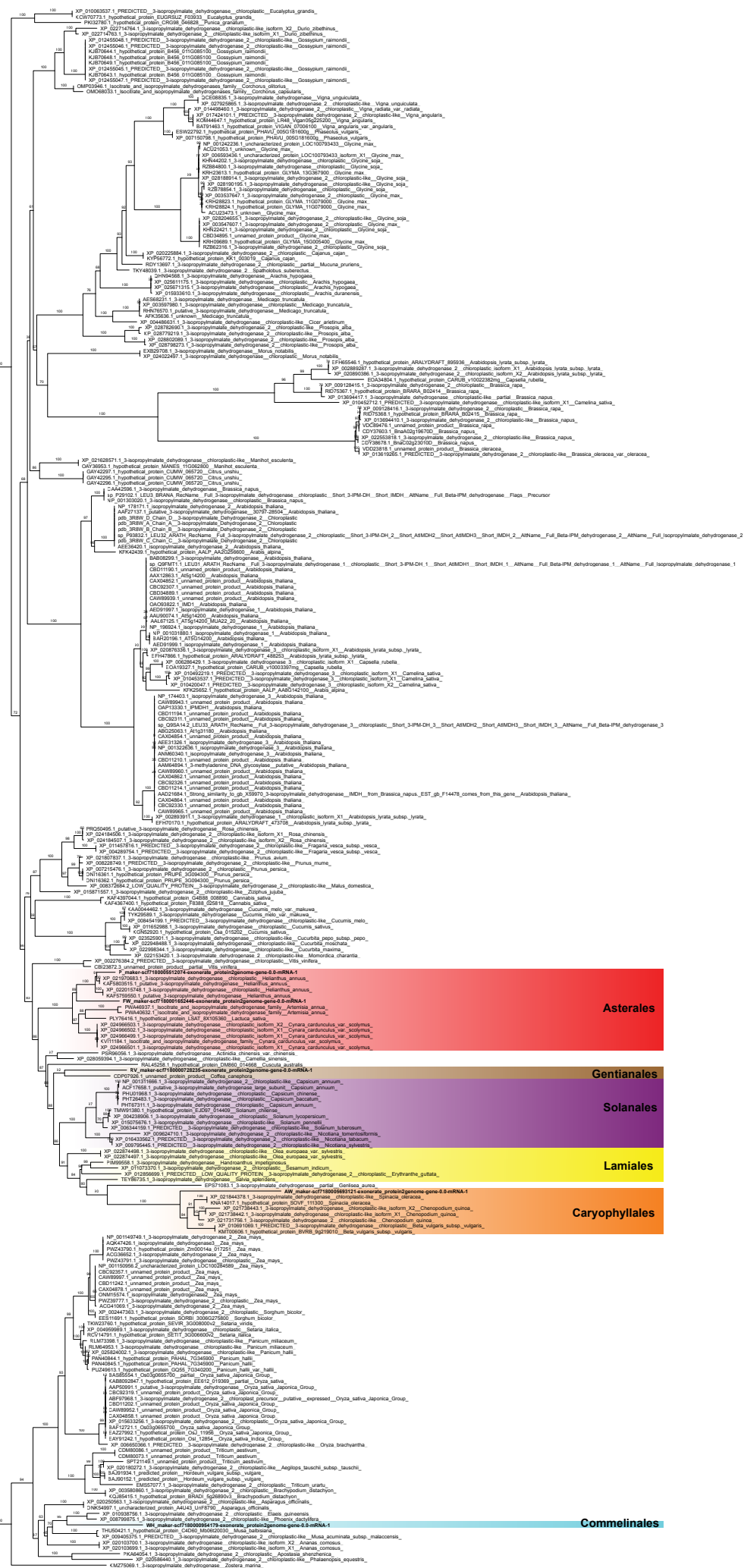

Supplementary Figure 11. Phylogenetic tree of plant taxa based on IMDH protein sequences.

Supplement: Supplementary file 11 [file Image_11.PDF]

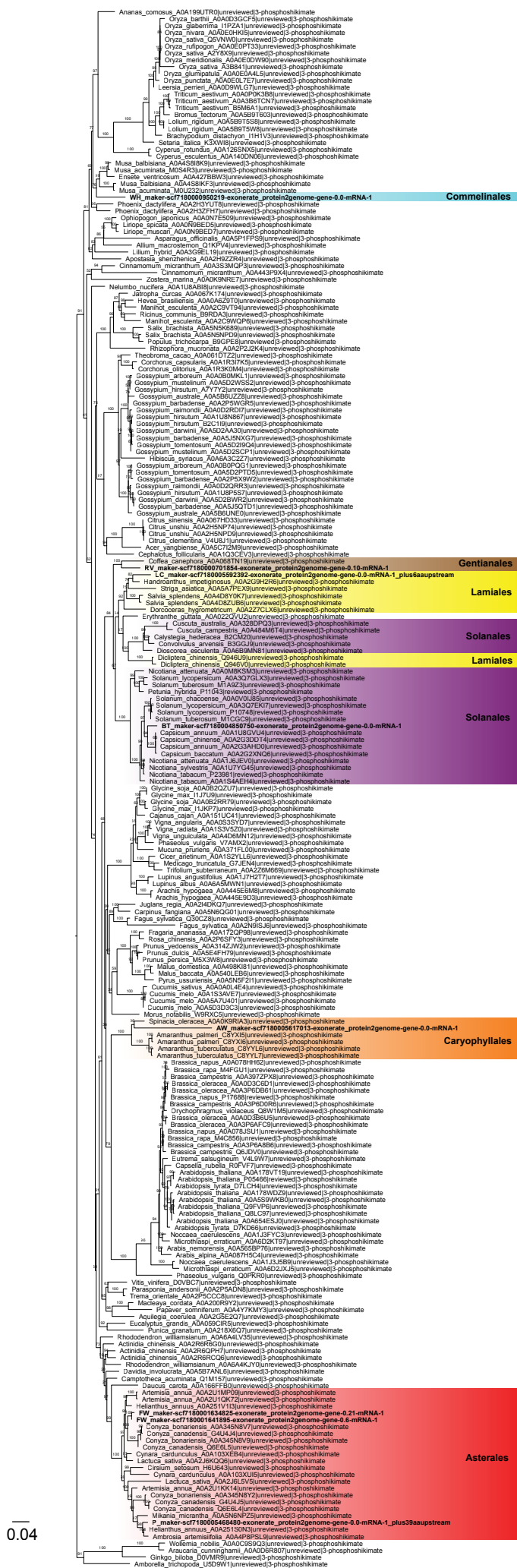

Supplementary Figure 12. Phylogenetic tree of plant taxa based on AroA protein sequences.

Supplement: Supplementary file 12 [file Image_12.PDF]

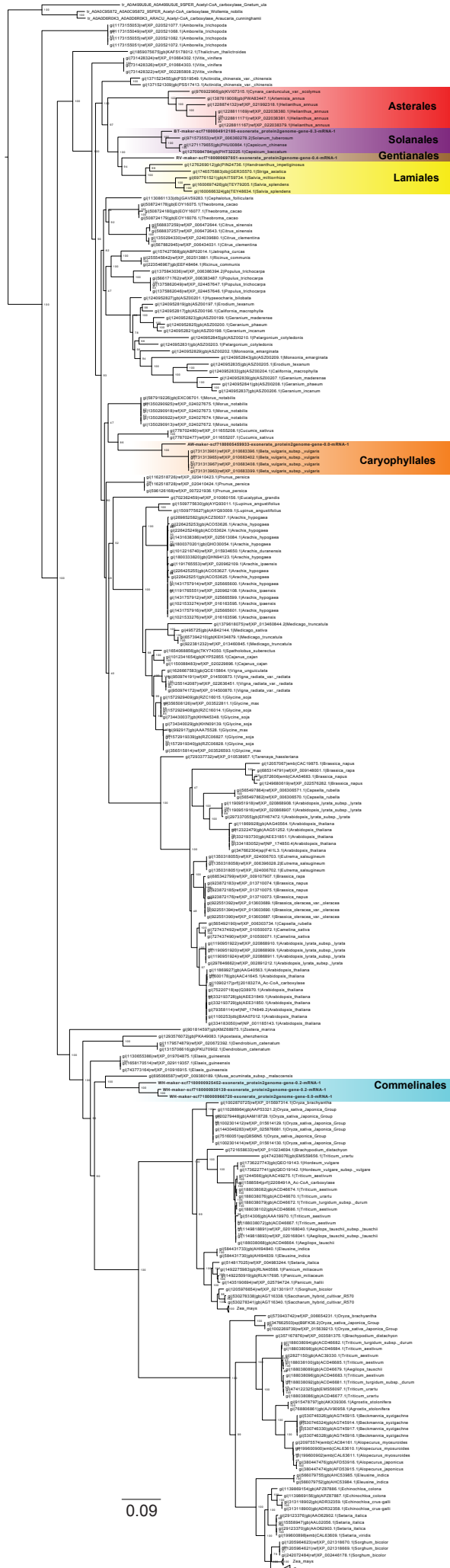

Supplementary Figure 13. Phylogenetic tree of plant taxa based on AccA protein sequences.

Supplement: Supplementary file 13 [file Image_13.PDF]

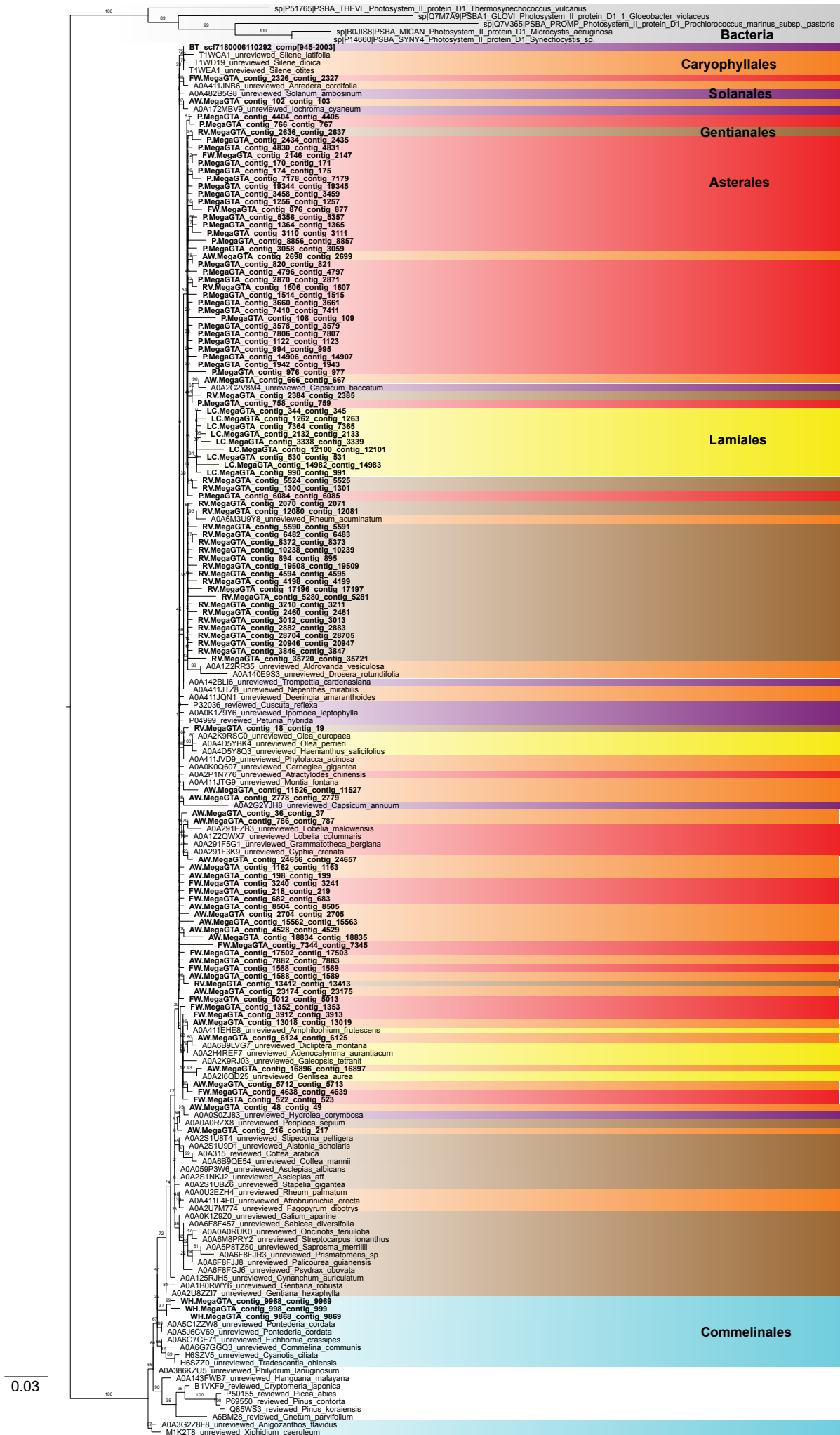

Supplementary Figure 14. Phylogenetic tree of plant taxa based on D1 (psbA) protein sequences.

Supplement: Supplementary file 14 [file Image_14.PDF]
